# Increased suicide risk among younger women in winter during full moon in northern Europe. An artifact or a novel finding?

**DOI:** 10.1038/s41380-022-01823-0

**Published:** 2022-10-20

**Authors:** Martin Plöderl, Joakim Westerlund, Sebastian Hökby, Gergö Hadlaczky, Michael Pascal Hengartner

**Affiliations:** 1 Department of Inpatient Psychotherapy and Crisis Intervention, University Clinic for Psychiatry, Psychotherapy, and Psychosomatics, Christian Doppler Clinic, Paracelsus Medical University, Ignaz-Harrer-Strasse 79, 5020 Salzburg, Austria; 2 Department of Clinical Psychology, Christian Doppler Clinic, Paracelsus Medical University, Salzburg, Austria; 3 National Centre for Suicide Research and Prevention, Centre for Health Economics, Informatics and Health Services Research, Stockholm Health Care Services, Stockholm, Sweden; 4 Department of Psychology, Stockholm University, Stockholm, Sweden; 5 National Centre for Suicide Research and Prevention; Department of Learning, Informatics, Management and Ethics, Karolinska Institutet, Stockholm, Sweden; 6 Department of Applied Psychology, Zurich University of Applied Sciences, Zurich, Switzerland

**Keywords:** Depression, Molecular biology

## Abstract

Available evidence suggests that there is no effect of moon phases on suicidal behavior. However, a Finnish study recently reported elevated suicide rates during full-moon, but only among premenopausal women and only in winter. This could not be replicated in an Austrian study and stirred a discussion about whether the Finnish finding was false-positive or if there are unaccounted moderator variables differing between Finland and Austria. The goal of the present study was to provide another replication with data from Sweden, which is geographically more comparable to Finland than Austria. We also investigated the discussed moderator variables latitude and nightly artificial brightness. There were 48,537 suicides available for analysis. The fraction of suicides during the full-moon quarter in winter did not differ significantly from the expected 25% among premenopausal women (23.3%) and in the full sample (24.7%). The incidence risk ratios for full moon quarter in Poisson regression models were 0.96 (95% CI: 0.90–1.02) for premenopausal women and 1.01 (95% CI: 0.99–1.04) for the full sample. According to Bayes-factor analysis, the evidence supports the null-hypothesis (no association) over the alternative hypothesis (some association). We found similar results when we split the data by latitude and artificial nightly brightness, respectively. In line with the Austrian study, there was no increase of suicides in Sweden among premenopausal women in winter during full-moon. The results from the Finnish study are likely false positive, perhaps resulting from problematic but common research and publication practices, which we discuss.

## Introduction

There is recurring interest in a putative effect of moon phases (i.e., synodic lunar cycle) on suicidal behavior despite a lack of convincing evidence. In fact, there is a wealth of research suggesting that the moon has no effect on suicidal behavior, and the issue was assumed to be settled long ago, in 1985, when a systematic review and meta-analysis reported a near perfect null-effect and no differences in relation to latitude, rurality, or gender [1]. A review from 1992 likewise concluded that “there is insufficient evidence for assuming a relationship between the synodic lunar cycle and completed or attempted suicide” [2]. Since then, the association between moon phases and suicidal behavior or psychiatric admissions was re-examined several times, without altering the widely accepted conclusion of a null-effect [3–5].

However, recently, a study from Northern Finland, district of Oulu, by Meyer-Rochow et al. (2021) (henceforth MR) found elevated suicide rates during full-moon, but only among premenopausal women and only in winter [6]. The finding was a statistically significant association between moon phase and suicide, which was discovered in subgroup analyses involving gender, age, and season. This data analysis seems to be exploratory, perhaps with post-hoc definitions of subgroups, increasing the risk of false-positive findings massively [7–10]. Therefore, Plöderl and Hengartner [11] (henceforth PH) argued that this spectacular and quite surprising finding was likely a statistical artifact, that is, a false-positive chance finding due to sampling variability. To bolster their argument, PH tried to replicate the findings from MR with higher powered data from Austria but found no increased suicide rate among premenopausal women during full moon phases in the winter season. In their response to this failed replication, anonymous reviewers and Meyer-Rochow et al. [12] argued that the discrepancies between the Austrian and Northern Finnish results could perhaps be explained by unaccounted moderator variables, for example, latitude, length of day/night, nightly artificial brightness, and Vitamin D deficiencies. In principle, their main argument was that the main finding reported by MR could not be replicated by PH with data from Austria, because Northern Finland was situated much further north than Austria and thus, the effect of full moon on premenopausal women during winter was only apparent in Northern Finland.

The main goal of our current study was to replicate the main findings of MR with data from Sweden, a country that is geographically more comparable to Northern Finland than Austria. Additionally, we also investigated the effect of some of the suggested moderator variables to explain the difference between the Austrian and Finnish study results, namely latitude and nightly artificial brightness. The results of the present study should thus also add novel insights pertaining to factors hypothesized to be involved in putative synodic lunar cycle effects on mental health.

## Materials and methods

### Suicide data

Suicide data was extracted from the pseudonymized Swedish Cause of death registry, provided by the Swedish National Board of Health and Welfare in 2022. Suicides were identified based on the related codes for intentional self-harm (X60–X84 or E950–E959) of the International Classification of Diseases (ICD), (8–10th revision). We only included cases where there was no doubt about the suicidal intent. The registry included all such cases between 1980 through 2021. Cases with missing data regarding exact death date and age were excluded in the main analyses, and cases with missing data about the municipality were excluded in the subgroup analyses about latitude and nightly brightness. Sweden has a total of 290 municipalities, and municipality data referred to where the deceased person resided at the time of death, according to the Swedish Tax Agency’s civil register. This variable provided the most fine-grained geographical information for suicides. In the protocol, we intended to use municipality where the suicide occurred, not the municipality of residency. This deviates from the protocol since we could obtain information for the municipality where the suicide occurred only for the years 2015–2021. However, this should not influence the results, as most suicides occur at the municipality of residency. For example, among individuals who died by suicide and resided in Stockholm, which is Sweden’s largest municipality, 80% died in Stockholm, while 13% died in a municipality close to Stockholm, and 7% died in a municipality further away. In Skellefteå, which is one of the largest municipalities included in our sensitivity analysis described below, 85% (*n* = 45) of the residents died by suicide in Skellefteå, while 2% (*n* = 1) died in a municipality very close to Skellefteå, and 13% (*n* = 7) of the cases had missing data about where the suicide took place.

### Lunar phases

Illumination of the moon for each day was calculated with R’s lunar Package [13] which provides a continuous measure of illumination for each date and also categories of lunar phases. The four-category measure cuts the illumination of the moon into quarters of equal size (full moon, waning moon, new moon, waxing moon); the eight-category measure cuts the illumination of the moon into eight categories of equal length. Similar to MR, we collapsed the four-level lunar-phase variable into a variable with three categories (full moon, new moon, waning/waxing moon combined). For sensitivity analysis, we used the eight-level lunar phases.

### Other variables

Seasons were defined in the same way as by MR: winter (November, December, January), spring (February, March, April), summer (May, June, July), and autumn (August, September, October). Age-groups were also defined in the same way as by MR, that is, women younger than 45 years were classified as premenopausal, while women 45 years or older were classified as postmenopausal. For latitudes of suicides, we used R’s swemaps package [14] to extract the geographic information about Swedens’s municipalities, that is, the polygon corresponding to each municipality. We then calculated the geographical center of each municipality and used it to calculate the latitude for each suicide (see Supplementary Figure). Nightly brightness for each of Sweden’s municipalities was calculated by linking the geographic information of the municipalities with the World Atlas of Artificial Night Sky Brightness [15]. We used the mean-value of the data-points extracted for each municipality (see Supplementary Figure).

### Statistical analyses

We used similar statistical methods as MR: χ^2^-tests were used to investigate gender differences for suicides and lunar phases. Multinomial tests were used to compare observed versus expected suicides in the three lunar phases, that is, we expected 25% suicides occurring during full moon, 25% for new moon, and 50% for waning/waxing moon within the subgroups (men, women, premenopausal women) in winter. We used similar Poisson-regression models as MR to investigate if daily suicides differed by lunar phases, with waning/waxing moon as the reference category, adjusted for seasons, with winter as the reference category. In addition to the statistical methods used by MR, we calculated Bayes-Factors (BF) to quantify how much the evidence supports the null hypothesis (zero difference in occurrence of suicides during full moon compared to other lunar phases) over the alternative hypothesis (more or less suicides during full-moon). For these binomial tests, we used the BayesFactor package [16]. We interpreted the BF according to Raftery (1995), as described by Wagenmakers [17]. Because of the multitude of analyses, statistically significant findings are expected to occur by chance alone (about 1 in 20 for a threshold of *p* < 0.05), thus we planned to use sequential Bonferroni alpha adjustments. We used R [18] for all analyses. The R-code is available via the Open Science Framework (OSF) https://osf.io/cfq3k/.

### Secondary analyses

For secondary analyses, we investigated the influence of two discussed potential moderator variables (latitude and nightly brightness) by running subgroup analyses. To do so, we dichotomized the variable on nightly brightness using the median value. For latitude, we used the mean-latitude of Sweden. This is a deviation from the protocol, where we stated to use the median value of the latitudes of the municipalities, but the median is then too far at the south of Sweden because most municipalities are located in the south. Consequently, this would prevent the estimation of differences between the northern and southern parts of Sweden. The subgroup analyses were conducted separately for the whole sample and for premenopausal women.

### Sensitivity analyses

We repeated the primary analysis restricted to municipalities with a latitude comparable to Oulu or even further north (to allow for comparability with MR). We decided to set the cut-off for latitude to also include Skellefteå municipality. This city is approximately 50 km south of Oulu but is one of the larger cities and thus will increase statistical power (see Supplementary Figure). In another sensitivity analysis we used data from March 1988 to June 2011, similar to MR. Finally, we repeated the analyses using eight levels of lunar phases, as done by PH.

### Pre-registration and availability of data

The study protocol was uploaded 2022-04-14 on the OSF https://osf.io/cfq3k/. The data used for this study was originally provided 2022-07-11 by the Swedish National Board of Health and Welfare, data-analyses. For ethical reasons, the raw data from the Swedish cause of death registry are not provided and was only accessible to the authors affiliated to the Swedish National Centre for Suicide Research and Prevention (JW, SH, GH). After uploading of the study-protocol, the R-code was developed by MP on a dummy data set of 1000 suicides and first applied by JW to the final data-set 2022-07-13. Methodological information to support replication by independent researchers is available from the corresponding author.

## Results

### Sample characteristics

There were 54,942 suicides occurring between 1980-01-01 and 2021-12-31. Of these, the day of death was known from 48,537 (88.3%), with 14,326 (29.5%) suicides by women and 34,211 (70.5%) suicides by men. There was missing data for age in 10 cases and for in municipality in 41 cases. There were 5580 suicides by premenopausal women available for the main analysis.

### Primary analysis: suicides by lunar phases

**Fig. 1:**
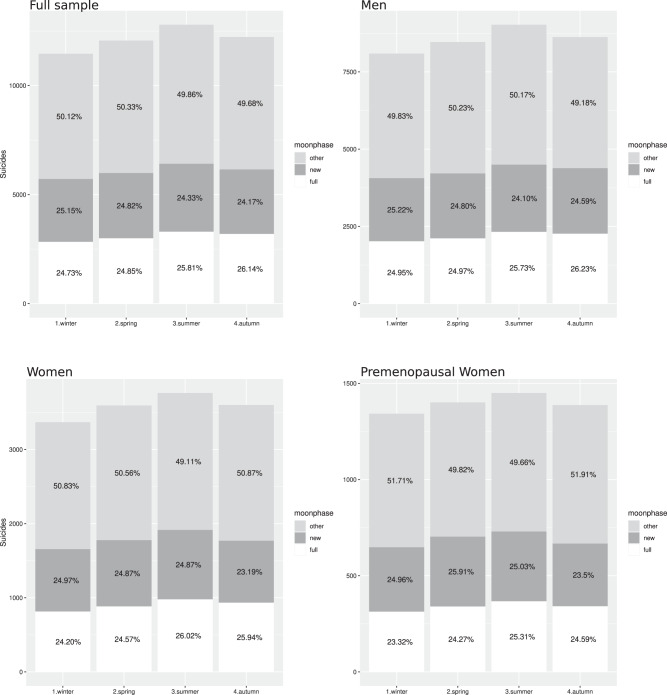
Distribution of suicides by season for the whole sample, men, women, and premenopausal women. The *y*-axis are absolute numbers of suicides. The numbers in the bars are percentages of observed suicides in the lunar phases. The expected percentages of suicides were 25% for the full moon quarter, 25% for the new moon quarter, and 50% for the other two quarters (waning/waxing) combined.

The distribution of suicides by lunar phases was close to the expected null-effect for the full sample, among men, women, and premenopausal women (Fig. 1, Table 1). In other words, the observed frequencies at full moon were close to the expected 25%. Our results showed that the fraction of suicides in winter during full moon was 24.7% in the full sample, 25.0% for men, 24.2% for women, and 23.3% for premenopausal women. This did not result in a significant gender difference (χ^2^ = 1.08, df = 2, *p* = 0.58). Within the subgroups, the distribution of suicides by lunar phases did not differ significantly from the expected distribution among men (χ^2^ = 0.22, df = 2, *p* = 0.90), women (χ^2^ = 1.33, df = 2, *p* = 0.51), or premenopausal women (χ^2^ = 2.30, df = 2, *p* = 0.32). Applying Bayesian binomial tests (full moon vs. other phases) resulted in BF = 0.03 for the full sample and for men (strong evidence for the null hypothesis), BF = 0.09 for women (positive evidence for the null hypothesis), and BF = 0.22 for premenopausal women (positive evidence for the null hypothesis).

**Table 1:** Observed and expected suicides for the full sample and for premenopausal women in the primary, secondary, and sensitivity analyses.

	Men and women					Premenopausal women				
Subgroup	*n*	*N*	%	*p*	BF	*n*	*N*	%	*p*	BF
Main analysis	2834	11,460	24.73	0.79	0.03	313	1342	23.32	0.32	0.22
Northern latitudes	271	1151	23.54	0.52	0.17	25	125	20.00	0.17	0.60
Southern latitudes	2409	9715	24.80	0.90	0.03	276	1160	23.79	0.64	0.14
Darker municipalities	1367	5569	24.54	0.46	0.05	180	769	23.41	0.58	0.18
Brighter municipalities	1313	5297	24.79	0.66	0.04	121	516	23.45	0.69	0.18
Latitude Oulo+	112	469	23.88	0.84	0.16	16	61	26.23	0.09	0.35
Time 1988/04–2011/06	1540	6125	25.14	0.74	0.04	165	686	24.05	0.29	0.13

*n* number of suicides during full moon in winter, *N* total number of suicides in winter, *p* *p*-value of the multinomial test, *BF* Bayes Factor of the binomial test, BF < 1 means that the evidence supports the null hypothesis (no effect of the moon) over the alternative hypotheses (some effect).

In Poisson regression models with daily suicide counts as outcome, adjusted for season, the incidence risk ratios (IRR) for full moon versus waning/waxing moon were IRR = 1.01 (95% CI: 0.99–1.04), *p* = 0.18 for the full sample, IRR = 1.02 (95% CI: 0.99–1.05), *p* = 0.12 for men, IRR = 1.00 (0.96–1.04), *p* = 0.98 for women, and IRR = 0.96 (0.90–1.02), *p* = 0.22 for premenopausal women (Tables 2–4). Suicide rates were significantly higher in spring, summer, and autumn, compared to winter in the models for men and women but not for the much smaller sample of premenopausal women. Among premenopausal women, there is strong evidence that a Bayesian Poisson regression model with only season as predictor is more supported by the data compared to a model also including lunar phases (BF = 0.01).

**Table 2:** Poisson regression results for the full sample and for premenopausal women in the primary, secondary, and sensitivity analyses.

	Men and women		Premenopausal women	
Subgroup	IRR [95% CI]	*p*	IRR [95% CI]	*p*
Main analysis	1.01 [0.99–1.04]	0.18	0.96 [0.90–1.02]	0.22
Northern latitudes	0.97 [0.90–1.04]	0.37	0.78 [0.63–0.97]	0.02
Southern latitudes	1.02 [1.00–1.04]	0.10	0.99 [0.92–1.06]	0.69
Darker municipalities	1.01 [0.98 - 1.04]	0.46	1.00 [0.92–1.09]	0.95
Brighter municipalities	1.02 [0.99–1.05]	0.28	0.91 [0.82–1.02]	0.09
Latitude Oulo+	0.99 [0.89–1.11]	0.89	0.84 [0.61–1.16]	0.28
Time 1988/04 - 2011/06	1.02 [0.99–1.06]	0.11	0.93 [0.84–1.02]	0.10

*IRR* incidence risk ratio, *95% CI* 95% confidence interval of the IRR, *p* *p*-value.

**Table 3:** Poisson regression results for men and for women, primary analysis.

Predictors	Men		Women	
	IRR [95% CI]	*p*	IRR [95% CI]	*p*
Intercept	0.88 [0.84–0.91]	<0.001	2.09 [2.04–2.14]	<0.001
Lunar phase				
Waning/waxing	Reference		Reference	
New	0.97 [0.94–1.01]	0.198	0.99 [0.97–1.02]	0.476
Full	1.00 [0.96–1.04]	0.984	1.02 [0.99–1.05]	0.117
Season				
Winter	Reference		Reference	
Spring	1.10 [1.05–1.15]	<0.001	1.08 [1.05–1.11]	<0.001
Summer	1.12 [1.07–1.17]	<0.001	1.12 [1.08–1.15]	<0.001
Autumn	1.07 [1.02–1.12]	0.005	1.07 [1.03–1.10]	<0.001

*IRR* incidence risk ratio, *95% CI* 95% confidence interval of the IRR, *p* *p*-value.

**Table 4:** Poisson regression results for premenopausal and postmenopausal women, primary analysis.

Predictors	Premenopausal women		Postmenopausal women	
	IRR [95%-CI]	*p*	IRR [95%-CI]	*p*
Intercept	0.35 [0.33–0.37]	<0.001	0.52 [0.50–0.55]	<0.001
Lunar phase				
Waning/waxing	Reference		Reference	
New	0.98 [0.92–1.05]	0.553	0.97 [0.92–1.02]	0.240
Full	0.96 [0.90–1.02]	0.215	1.03 [0.98–1.08]	0.311
Season				
Winter	Reference		Reference	
Spring	1.08 [1.00–1.16]	0.055	1.12 [1.05–1.19]	<0.001
Summer	1.08 [1.00–1.16]	0.041	1.14 [1.08–1.21]	<0.001
Autumn	1.03 [0.96–1.11]	0.388	1.09 [1.03–1.16]	0.004

*IRR* incidence risk ratio, *95%-CI* 95% confidence interval of the IRR, *p* *p*-value.

In contrast to our study, MR found that the proportion of suicides occurring during full-moon in winter was 24.3% among men but 40%, among women, resulting in a significant gender difference (*p* < 0.004). MR also found a statistically significant IRR = 1.28 (1.04–1.57) for women and IRR = 1.35 (1.01–1.80) for premenopausal women (calculated from their Table 1).

### Secondary analysis: latitude and nightly artificial brightness as moderating variables

Separate analyses of suicides occurring in municipalities in the northern and southern latitudes, or in municipalities with darker or brighter nightly artificial brightness showed that the observed proportion of suicides in winter during full moon was close to the expected 25%. This did not result in any statistically significant difference (Tables 1 and 2) with one exception: for premenopausal women in northern municipalities, there were *fewer* suicides during full moon in winter in the regression analysis adjusted for season (IRR = 0.78, 95% CI: 0.63–0.97, *p* = 0.02), however, this is not significant when accounting for multiple testing (the required p-level for significance after correction is *p* < 0.007). Moreover, the BF suggested that the evidence is consistently favoring the null-hypothesis (no difference) over the alternative hypothesis (any difference). The evidence was strong for the full sample, and positive for premenopausal women.

### Sensitivity analysis

When restricting the analysis to suicides occurring in municipalities with latitude comparable to Oulu or even further north, the observed proportion of suicides during full moon in winter was again close to the expected 25%, the difference being not statistically significant (Tables 1 and 2). The BF indicates that the evidence was in favor or the null-effect (strong for the full sample and weak for premenopausal women). Another sensitivity analysis, restricting the data to March 1988 to June 2011 as done by MR, lead to very similar results when compared to the main analysis. Finally, using eight levels of lunar phases with new-moon as reference category in a regression model for premenopausal women likewise showed no significant effect, IRR = 0.93 (0.74–1.16), *p* = 0.51.

## Discussion

In our study based on the Swedish cause of death registry, we could not replicate the finding by MR of an increased rate of suicides in premenopausal women during full moon in winter [6]. In contrast, we found that the observed proportion of suicides in winter was very close to the expected 25% in the full-moon quarter in the full sample, among men and women, and among premenopausal women. Bayesian analyses suggested that the evidence is more in favor of the null hypotheses (no change of suicides during full moon in winter) than the alternative hypothesis (some change). We also explored latitude and nightly artificial brightness, two moderating variables which were suggested to explain the difference between the Finnish study and a previous Austrian replication study by PH [11], which also failed to replicate the findings by MR. We found null-findings in both northern and southern municipalities as well as in both municipalities with darker and brighter artificial nightly brightness. There was one exceptional significant result in the regression analysis for premenopausal women in northern municipalities suggesting *lower* suicide rates during full moon, but significance was lost after adjusting the significance level alpha for multiple testing. Finally, we found null-findings in three sensitivity analyses where the data was restricted to the same latitude (or even further north) as in the sample by MR, using the same time-frame as MR, and where an eight-level lunar phase was used.

Thus, based on our findings, together with the previous failed Austrian replication study, it can be concluded safely that suicide rates do not vary according to lunar phases in winter (or other seasons), neither in premenopausal women, nor in women of all ages and in men. Subgroup analyses suggested that moderating variables such as latitude and nightly artificial brightness do not influence the association between lunar phases and suicide rates. Furthermore, the present study and the previous Austrian study were pre-specified replication studies, preventing p-hacking, for example by defining arbitrary subgroups or outcome measures post hoc. The statistical power was much larger both in the present study and in the Austrian study compared to the study by MR, increasing the chance that even smaller effects are detected.

There are several implications worth discussing. Our Swedish study, together with the previous Austrian replication study found near-perfect null findings, since the observed proportion of suicides during the full moon quarters were very close to the expected 25%. These findings, together with the predominating null-findings reported in the literature strongly suggest that the findings by MR were false-positive. This is not surprising because it is common that unexpected published study findings are false positives [8]. We think that the study by MR is a case in point example of dynamics related to the “generation” of false-positive findings and how these are treated in the scientific community and publication systems.

We can only speculate about reasons why MR found the exceptional, most likely false positive finding. It is well-known that the creation of many subgroups in a data-set increases the risk that a result will be statistically significant by chance alone due to sampling variability, even when there is a perfect null-association in the underlying population [19]. This is why the correction of the level of significance is commonly used to guard against false positives in subgroups. Furthermore, it is unclear why MR defined some of their subgroups and outcome variables the way they did, since hardly any justification was given in the paper. For example, their definition of winter is questionable because it included November, December, and January. Astronomically, winter starts at December 21 in the northern hemisphere, therefore winter might as well be defined from December to February, which would also be in accordance with the meteorological months of winter. Alternatively, MR could have used the precise range from December 21 +/− 45.7 days (November 5 to February 4). Similarly, instead of a splitting the lunar phase into quarters (and collapsing the waning/waxing lunar phases into one), a more fine-grained splitting could have been used, or even a continuous measure with the least loss of information, as used by PH in an additional analysis [11]. Another point of criticism is the cut-off for menopause, which starts, at average, between 49 and 52 years [20], whereas MR classified women aged 44 or younger as premenopausal. Moreover, is not clearly stated in the paper by MR whether these subgroups were planned a-priori, or exploratory strategies were involved. There is nothing wrong with exploratory analysis. However, if an exceptional finding occurs only in a specific subgroup and in the absence of plausible causal mechanisms, sensitivity analyses may be necessary to check for robustness. At the very least, as PH pointed out, such findings should be explicitly declared as explorative and possibly false positive, with the need for replication, to avoid creating the illusion that the analyses were testing a-priori hypotheses. Whether intentional or not, failing to point out explorative strategies and reporting post-hoc analyses as if they were planned a-priori constitutes “p-hacking”, “fishing for significance” [7, 9, 17], or “HARKing” (hypothesizing after the results are known) [21, 22].

It is well known that the problematic research and publication practices discussed above led to a replication-crisis in science because many research findings could not be replicated, and the percentage of null-findings sharply increased in replication and pre-registered research [23] and by independent researchers [24]. In this regard, it is not surprising that despite a likely absent effect of the moon on suicidal behavior, there are a few publications reporting significant associations between lunar phases and mental health problems. The notion that these findings were likely false positive is supported by the fact that the associations were again mostly found in certain subgroups and the studies were not pre-registered. Furthermore, to our knowledge, none of these studies were successfully replicated, or perhaps failed replications were never published. These exceptional findings also stand in stark contrast to the predominating studies finding no association between lunar phases and suicide risk. But even without selective publication and p-hacking, sampling variability must lead to a fraction of statistically significant findings by chance alone, even if there is no true association in the underlying population, because this is the very nature of sampling and null-hypothesis testing. The risk that significant results are false positive is even higher under cirumstances where the prior probability of a hypothesis being true is low [8], which is certainly the case for the association between lunar phases and suicide. Even without these considerations, MR’s finding that there is an effect of IRR = 1.35 (1.01–1.80) among premenopausal women should have raised doubts, because the point estimate is a substantial effect in suicidology and the lower bound is very close to the null-effect.

The generation of additional hypotheses to explain a failed replication is sometimes not only intended to improve the scientific process but to immunize against the rejection of a hypothesis. It can always be claimed that any replication differs in some way from the original study and thus always be considered as insufficient. How many more failed replications are then needed to consider a finding as false-positive? Despite the predominating null-results in previous studies and the null-finding in the Austrian study, MR suggested “It is necessary to guard against a well-documented reluctance to embrace novel suggestions, ideas and findings that depart from convention” (Semmelweis-Reflex) [12]. We do not deny that there is an influence of the moon on some biological mechanisms in certain species [25]. We also agree that we have to be open for novel and unexpected findings. According to Munafo et al., “however, a major challenge for scientists is to be open to new and important insights while simultaneously avoiding being misled by our tendency to see structure in randomness. The combination of apophenia (the tendency to see patterns in random data), confirmation bias (the tendency to focus on evidence that is in line with our expectations or favored explanation) and hindsight bias (the tendency to see an event as having been predictable only after it has occurred) can easily lead us to false conclusions” [22]. In the present study, we both tried to address the criticism and additional hypotheses formulated by Meyer-Rochow [12] and peer-reviewers of PH as well as the obvious need for replication to uncover a false positive finding. Thus, we hope that our study not only shed light on the putative effect of the moon on suicidal behavior, which is most likely absent, but also on the dynamics related to novel and unexpected findings in science.

## Supplementary information

Supplementary Figure

## Data Availability

Data cannot be made publicly available (see main text for details).

## References

[1] Rotton J, Kelly IW (1985). Much ado about the full moon: A meta-analysis of lunar-lunacy research. Psychol Bull.

[2] Martin SJ, Kelly IW, Saklofske DH (1992). Suicide and lunar cycles: a critical review over 28 years. Psychol Rep..

[3] Gupta R, Nolan DR, Bux DA, Schneeberger AR. Is it the moon? Effects of the lunar cycle on psychiatric admissions, discharges and length of stay. Swiss Med Wkly. 2019. 10.4414/smw.2019.20070. 10.4414/smw.2019.20070 31012946

[4] McLay RN, Daylo AA, Hammer PS (2006). No effect of lunar cycle on psychiatric admissions or emergency evaluations. Mil Med.

[5] Biermann T, Estel D, Sperling W, Bleich S, Kornhuber J, Reulbach U (2005). Influence of lunar phases on suicide: the end of a myth? A population‐based study. Chronobiol Int.

[6] Meyer-Rochow VB, Hakko T, Hakko H, Riipinen P, Timonen M (2021). Synodic lunar phases and suicide: based on 2605 suicides over 23 years, a full moon peak is apparent in premenopausal women from northern Finland. Mol Psychiatry.

[7] Forstmeier W, Wagenmakers E-J, Parker TH (2017). Detecting and avoiding likely false-positive findings - a practical guide. Biol Rev.

[8] Ioannidis JPA (2005). Why most published research findings are false. PLoS Med.

[9] Wicherts JM, Veldkamp CLS, Augusteijn HEM, Bakker M, van Aert RCM, van Assen MALM (2016). Degrees of freedom in planning, running, analyzing, and reporting psychological studies: a checklist to avoid p-Hacking. Front Psychol..

[10] Wagenmakers E-J, Wetzels R, Borsboom D, van der Maas HLJ, Kievit RA (2012). An agenda for purely confirmatory research. Perspect Psychol Sci.

[11] Plöderl M, Hengartner MP (2021). Moon and suicide: a true effect or a false-positive finding?. Mol Psychiatry.

[12] Meyer-Rochow VB, Hakko T, Hakko H, Riipinen P, Timonen M (2021). Difficulties that unexpected results face to be accepted: suicide and the moon. Mol Psychiatry.

[13] Lazardis E. Lunar: Lunar Phase & Distance, Seasons and Other Environmental Factors. Version 0.1-04.

[14] Reinholdsson T. Swemaps: Swedish map data for ggplot and leaflet in R. R package version 1.0. 2015.

[15] Falchi F, Cinzano P, Duriscoe D, Kyba CCM, Elvidge CD, Baugh K, et al. Supplement to: The New World Atlas of Artificial Night Sky Brightness. 2016. 10.1126/sciadv.1600377 PMC4928945 27386582

[16] Morey R, Rouder J. BayesFactor: Computation of Bayes Factors for Common Designs. R package version 0.9.12-4.3 2021.

[17] Wagenmakers E-J (2007). A practical solution to the pervasive problems ofp values. Psychonomic Bull Rev.

[18] R Core Team. R: A language and environment for statistical computing. Vienna, Austria: R Foundation for Statistical Computing; 2022.

[19] Simmons JP, Nelson LD, Simonsohn U (2011). False-positive psychology: undisclosed flexibility in data collection and analysis allows presenting anything as significant. Psychol Sci.

[20] Takahashi TA, Johnson KM (2015). Menopause. Med Clin North Am.

[21] Kerr NL (1998). HARKing: hypothesizing after the results are known. Pers Soc Psychol Rev.

[22] Munafò MR, Nosek BA, Bishop DVM, Button KS, Chambers CD, Percie du Sert N (2017). A manifesto for reproducible science. Nat Hum Behav.

[23] Allen C, Mehler DMA (2019). Open science challenges, benefits and tips in early career and beyond. PLoS Biol.

[24] Mac Giolla E, Karlsson S, Neequaye DA, Bergquist M. Evaluating the replicability of social priming studies. PsyArXiv. 2022.

[25] Bevington M (2015). Lunar biological effects and the magnetosphere. Pathophysiology.

