# Supplementary figures and images for: Increased suicide risk among younger women in winter during full moon in northern Europe. An artifact or a novel finding?
Source: Mol Psychiatry. 2022 Oct 20;28(2):901–7. doi: 10.1038/s41380-022-01823-0 (PMC9908532; doi:10.1038/s41380-022-01823-0)

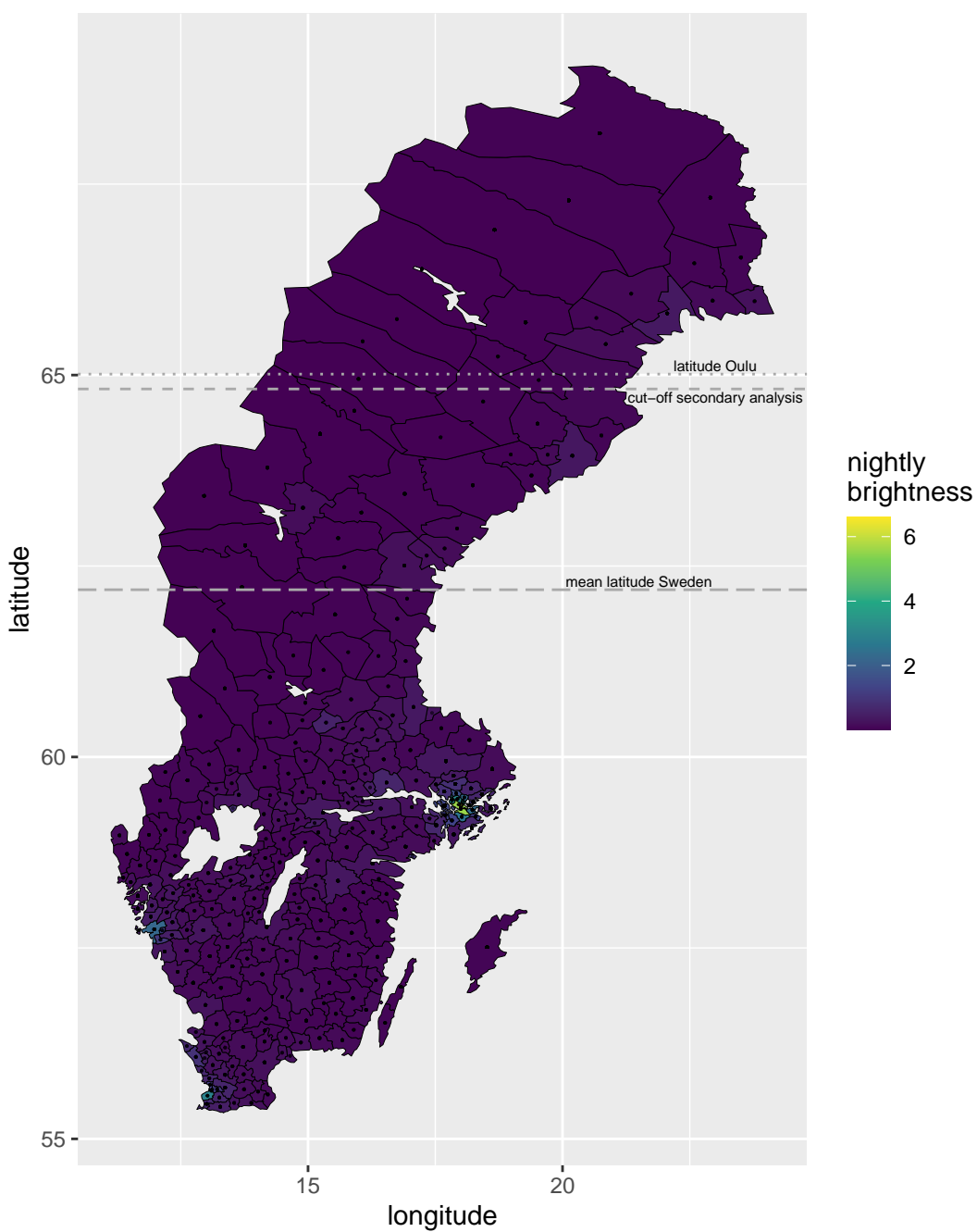

Supplement: Supplementary file 1 — Supplementary Figure [file 41380_2022_1823_MOESM1_ESM.pdf]
